# Growth of sillenite Bi_12_FeO_20_ single crystals: structural, thermal, optical, photocatalytic features and first principle calculations

**DOI:** 10.1038/s41598-020-78598-3

**Published:** 2020-12-16

**Authors:** Durga Sankar Vavilapalli, Ambrose A. Melvin, F. Bellarmine, Ramanjaneyulu Mannam, Srihari Velaga, Himanshu K. Poswal, Ambesh Dixit, M. S. Ramachandra Rao, Shubra Singh

**Affiliations:** 1 Crystal Growth Centre, Anna University, Chennai, 600025 India; 2 University of Bordeaux, ISM UMR CNRS 5255, Bordeaux INP, ENSCBP, 16 Avenue Pey Berland, Bordeaux, 33607 Pessac France; 3 Nano Functional Materials Technology Centre, Materials Science Research Centre and Department of Physics, Indian Institute of Technology Madras, Chennai, 600036 India; 4 Division of Physics, Department of Science and Humanities, Vignan’s Foundation for Science, Technology and Research, Guntur, 522213 India; 5 High Pressure and Synchrotron Radiation Physics Division, Bhabha Atomic Research Centre, Mumbai, 400085 India; 6 Department of Physics and Centre for Solar Energy, Indian Institute of Technology Jodhpur, Jodhpur, 342 037 India

**Keywords:** Chemistry, Energy science and technology, Materials science, Physics

## Abstract

Ideal sillenite type Bi_12_FeO_20_ (BFO) micron sized single crystals have been successfully grown via inexpensive hydrothermal method. The refined single crystal X-ray diffraction data reveals cubic Bi_12_FeO_20_ structure with single crystal parameters. Occurrence of rare Fe^4+^ state is identified via X-ray photoelectron spectroscopy (XPS) and X-ray absorption spectroscopy (XAS). The lattice parameter (*a*) and corresponding molar volume (*V*_*m*_) of Bi_12_FeO_20_ have been measured in the temperature range of 30–700 °C by the X-ray diffraction method. The thermal expansion coefficient (*α*) 3.93 × 10^–5^ K^−1^ was calculated from the measured values of the parameters. Electronic structure and density of states are investigated by first principle calculations. Photoelectrochemical measurements on single crystals with bandgap of 2 eV reveal significant photo response. The photoactivity of as grown crystals were further investigated by degrading organic effluents such as Methylene blue (MB) and Congo red (CR) under natural sunlight. BFO showed photodegradation efficiency about 74.23% and 32.10% for degrading MB and CR respectively. Interesting morphology and microstructure of pointed spearhead like BFO crystals provide a new insight in designing and synthesizing multifunctional single crystals.

## Introduction

Sillenite is a rich family consisting of more than 60 individual compounds and solid solutions. Sillenite structured complex metal oxides exist with general formula Bi_12_MO_20_, where M is a tetravalent cation (M: Si, Ti, Ge etc.) constituting discrete MO_4_ tetrahedra, separated by Bi-O frameworks to form a body centered cubic lattice with space group *I23*^1–6^. These Bi rich compounds are attractive materials for practical applications owing to their photoconductive, photorefractive, piezoelectric, pyroelectric and nonlinear optical (NLO) properties^7^. The catalytic properties encourage potential use of these compounds for photocatalytic applications in organic effluent treatment and water splitting. One such material, Bi_24_Ga_2_O_30_, plays the role of an efficient photocatalyst in degrading Methylene blue^8^. Few sillenite compounds also possess nonlinear optical (NLO) properties with a potential to be used further for second harmonic generation (SHG) applications. Such wide scale potential applications have led researchers to propose sillenite structures based on transition-metal ions, like Mn and Fe, exploring the role of these multivalent cations. The fact that these cations can exhibit tetravalency, as in Mn^4+^ and Fe^4+^, has led us to investigate them further for their influential properties. Tetravalency of Fe and Mn is rarely observed in perovskites and related complex oxides. These tetravalent cations (Fe^4+^ and Mn^4+^) form an ideal sillenite structure (e.g. Bi_12_FeO_20_, Bi_12_MnO_20_ etc.)^9^. Until now researchers have investigated and proposed Fe based non-ideal sillenite single crystals such as Bi_12_Fe_0.63_O_18.945_ and Bi_25_FeO_40,_ constituting Fe^3+^ oxidation states with Bi-rich content which can be used for photoactive applications^10, 11^. These sillenite compounds often occur as intermediate products in the preparation of BiFeO_3_. Proximity to the class of Bi-rich iron-based oxides makes these compounds attractive with wide interests. As compared to widely used UV operated photocatalysts such as ZnO and TiO_2_, sillenites offer optical band gap values between 2.5 and 2.8 eV which lie in the visible region of solar spectra making them photoactive with potential applications in water splitting and dye degradation under visible light^12, 13^. Not much research has been carried out on related ideal sillenite type Bi_12_FeO_20_ compound.

In this communication, to the best of our knowledge, we report for the first time a spearhead like ideal sillenite structured Bi_12_FeO_20_ single crystals_._ Non-ideal Fe based Bi-rich sillenite materials, such as crystallites of Bi_12_Fe_0.63_O_18.945_ and Bi_25_FeO_40_ single crystals, have been investigated before^14, 15^. An ideal Sillenite structure of polycrystalline Bi_12_FeO_20_, synthesized at elevated temperatures by typical solid state reaction method, has also been reported by Elkhoun et al^16^. First report on low temperature synthesis of ideal structured Bi_12_FeO_20_ single crystals with rare Fe^4+^ state via inexpensive hydrothermal process is revealed here, along with systematic characterization and application related studies.

## Experimental

### Crystal growth

High pure Bi(NO_3_)_3_.5H_2_O and Fe(NO_3_)_3_.9H_2_O as starting precursors and 0.2 M concentration each were dissolved in 50 ml of deionized water. Few drops of HNO_3_ were added to get clear transparent solution. After vigorous stirring, 50 g of KOH was added and the temperature of solution was cooled down to room temperature before transferring it into a 100 mL Teflon-lined autoclave, upto 70% of its maximum capacity. Crystallization takes place at 200 °C for 72 h. Post this, the autoclave was cooled and depressurized, product was washed with distilled water, sonicated and then the sample was harvested as fine, reddish brown crystals.

### Characterization

For structural analysis, Single crystal X-ray diffraction (SC-XRD, Bruker Kappa ApexII) was performed and the lattice parameters were obtained by refining Single Crystal XRD data by SHELXTL refining software^17, 18^. High Resolution transmission electron microscopic (HRTEM) images and selected area electron diffraction patterns (SAED) were recorded on an FEI Tecnai TF-20 operating at 200 kV. X-ray photoelectron spectroscopic (SPECS GmbH, Germany) measurements were conducted to investigate the Fe and Bi oxidation states. X-ray absorption spectroscopy (XAS) measurements were carried out to examine the valence state of Fe in Bi_12_FeO_20_(BFO). The experiments were performed at the Energy-Scanning EXAFS beamline (BL-9) at the Indus-2 Synchrotron Source (2.5 GeV, 200 mA), Raja Ramanna Centre for Advanced Technology (RRCAT), Indore, India^19, 20^.

### Synchrotron based powder X-ray diffraction measurements

Synchrotron based powder X-ray diffraction measurements were carried out on well ground powder samples of Bi_12_FeO_20_ single crystals at Extreme Conditions X-ray diffraction (EC-XRD) beamline (BL-11) at Indus-2 synchrotron source, Raja Ramanna Centre for advanced Technology (RRCAT), Indore, India. High temperature measurements were carried out on STOE high temperature attachment 0.65.3 with Eurotherm 2416 controller. Desired wavelength (0.6285 Å) for ADXRD diffraction experiments was selected from white light from the bending magnet using a Si(111) channel cut monochromator. The monochromatic beam is then focused on to the sample with a Kirkpatrick-Baez mirror or K-B mirror. A MAR345 image plate detector (which is an area detector) was used to collect 2-dimensional diffraction data. Sample to detector and the wavelength of the beam were calibrated using NIST standards LaB_6_ and CeO_2_. Calibration and conversion/integration of 2D diffraction data to 1D, intensity vs 2θ, was carried out using FIT2D software^21, 22^.

### Electronic structure calculations

The density functional calculations are carried out under full potential linear augmented plane wave (FP-LAPW), as implemented in Wien2K^23^. The modified Becke-Johnson parameterization is used as an exchange correlation function^24^. The unit cell is divided in muffin tin region (with R_mt_ as radius) and interstitial region (IR). The muffin tin radii for bismuth, iron and oxygen atoms are chosen in such a way that there is no overlap among different atomic elements. The plane wave cut off parameters R_mt_ × K_max_ = 7 and G_max_ = 12 are used for structural, electronic and optical properties of Bi_12_FeO_20_. The maximum value of *l* (*l*_max_) is considered 10 and cut-off energy is at – 7.0 Ry, defining the separation between the core and valence states. The self-consistent calculations are carried out under total energy convergence of∼ 0.001 Ry. A large plane wave cut-off of 150 Ry is used throughout the calculation and initially 125 K-points are considered in Brillouin zone for optimization while 1000 K points are used for computing the other properties like electronic and optical properties of the material.

## Results and discussion

**Figure 1:**
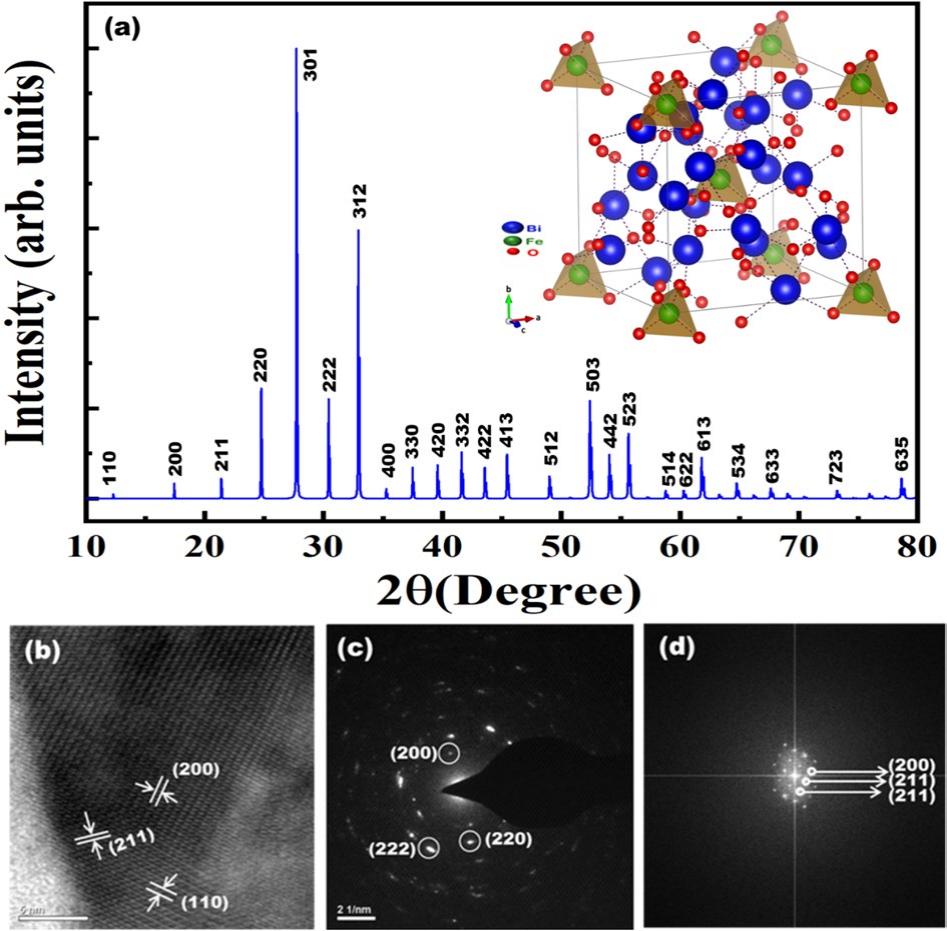
(**a**) Single crystal XRD pattern of Bi_12_FeO_20_ (inset crystal structure of Bi_12_FeO_20_ comprising of body-centered cubic FeO_4_ tetrahedra) (**b**) HR-TEM image of BFO (**c**) SAED pattern of BFO (**d**) Corresponding fast fourier transform (FFT) image.

Single crystal X-ray diffraction data for Bi_12_FeO_20_ (BFO) crystals were measured at 296 K using a Bruker Kappa Apex II with a wavelength of MoKα radiation of 0.7107 Å. The structure was refined using SHELXTL refining software^17, 18^_._ The refined XRD pattern and crystal structure is shown in Fig. 1a. The lattice parameters of BFO are a, b and c = 10.1713(10) Å and α, βand γ = 90° and the cell volume is V = 1052.28(3) Å^3^. Single crystal structure refinement data is presented in Table S1 and the refined data indicates the formation of Bi_12_FeO_20_ with body centered cubic crystal system and *I23* space group. The optimization of crystal structure is performed and the calculated variation of volume versus energy is shown in Fig. S1. A clear minimum noticed at − 523,513.3 eV, correspond to the minimum energy structure. The computed lattice parameter is *a* = 10.2707 (19.4154 Bohr) Å, and is in good agreement with experimental refined XRD data.

Figure 1b shows the HRTEM image of BFO single crystals, with clear resolved crystalline domains and an interplanar distance 0.71 nm, 0.5 nm and 0.415 nm corresponding to (110, (200) and (211) planes respectively. In Fig. 1c the SAED pattern reveals the presence of (200), (220) and (222) planes. Fast Fourier transform of HRTEM image gives an evidence of the crystallinity of BFO as shown in Fig. 1d.

**Figure 2:**
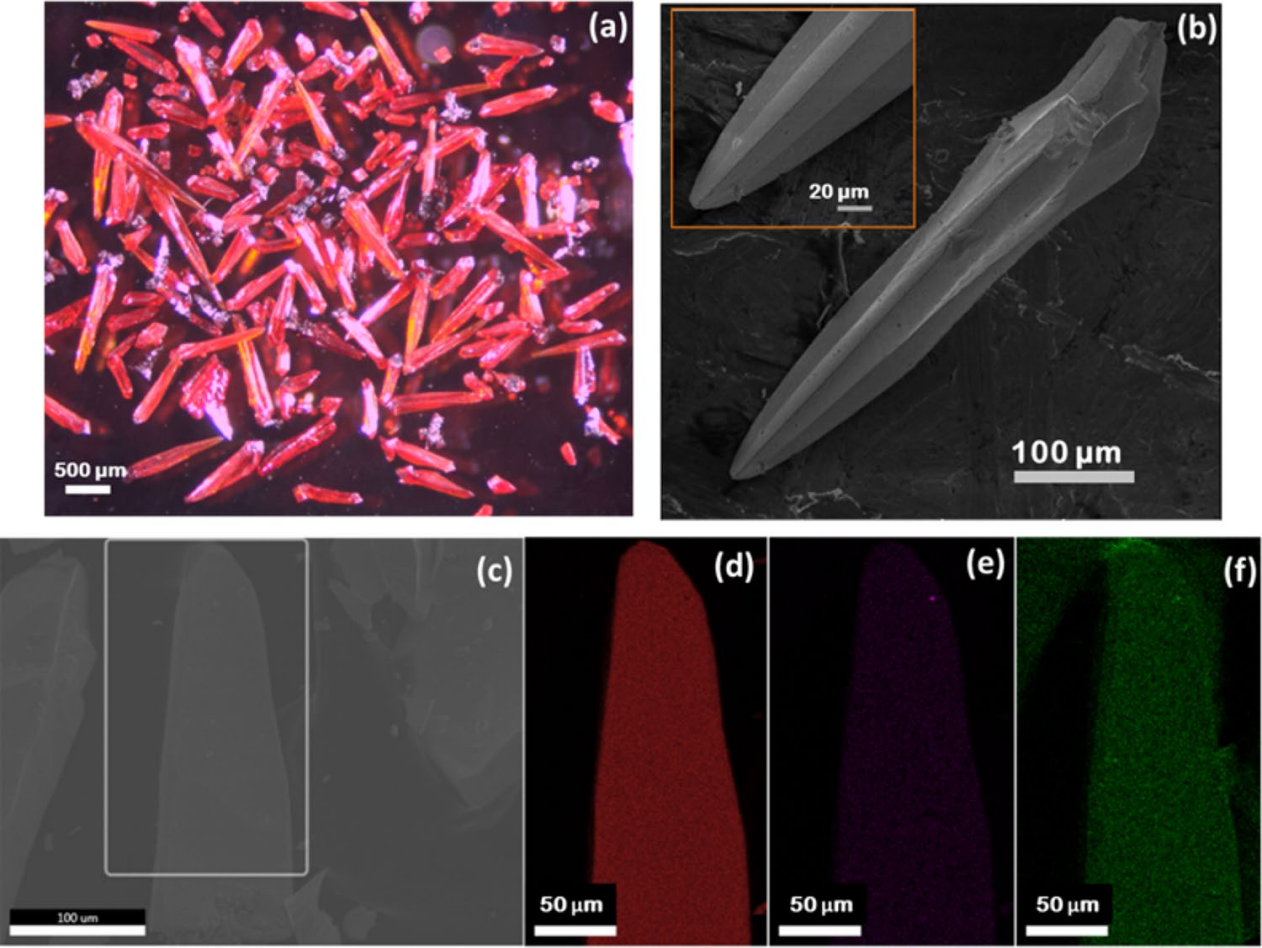
(**a**) Optical microscope image of Bi_12_FeO_20_ crystals (**b**) SEM image and the EDS elemental mapping of the BFO crystals (**c**) SEM image of EDS mapped area (**d**) Bi, (**e**) Fe, (**f**) O elements.

Optical and scanning electron microscopy images of BFO in Fig. 2a,b reveal interesting spearhead like morphology with an average size ~1 mm. EDS mapping of BFO crystals showed a uniform distribution of Bi, Fe and O throughout the crystal Fig. 2c–f.

**Figure 3:**
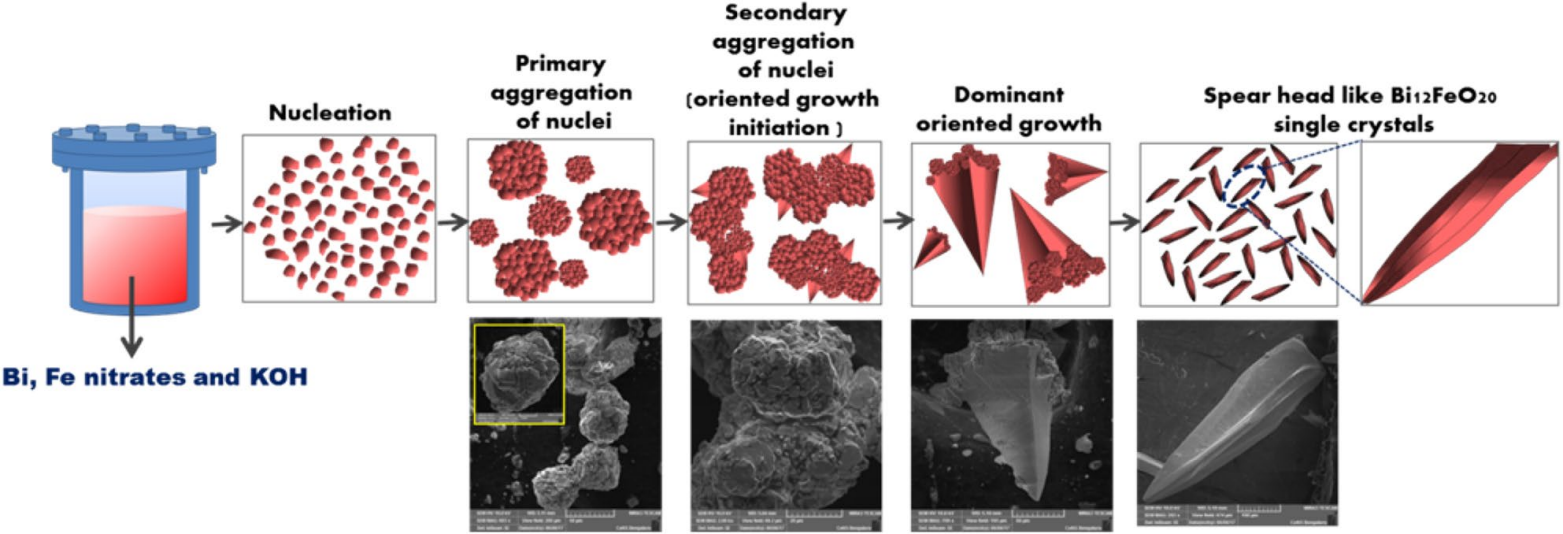
Schematic illustrations of hydrothermally grown spear head like Bi_12_FeO_20_ single crystals with corresponding SEM images.

A possible growth mechanism of spearhead like BFO crystals based on microscopic revelations has been illustrated and represented with a schematic in Fig. 3. SEM images recorded at various stages of crystallization of BFO proves that the growth mechanism involves various steps starting from nucleation to single crystal formation. During the hydrothermal growth at applied temperature and autogenerated pressure, supersaturation takes place and initiates nucleation in the precursor solution. An increase in growth time tends to aggregate the nuclei to form clusters. This step is followed by secondary aggregation of clusters initiating oriented growth which is desirable in single crystal. At an optimized growth time and temperature complete growth of crystal takes place.

**Figure 4:**
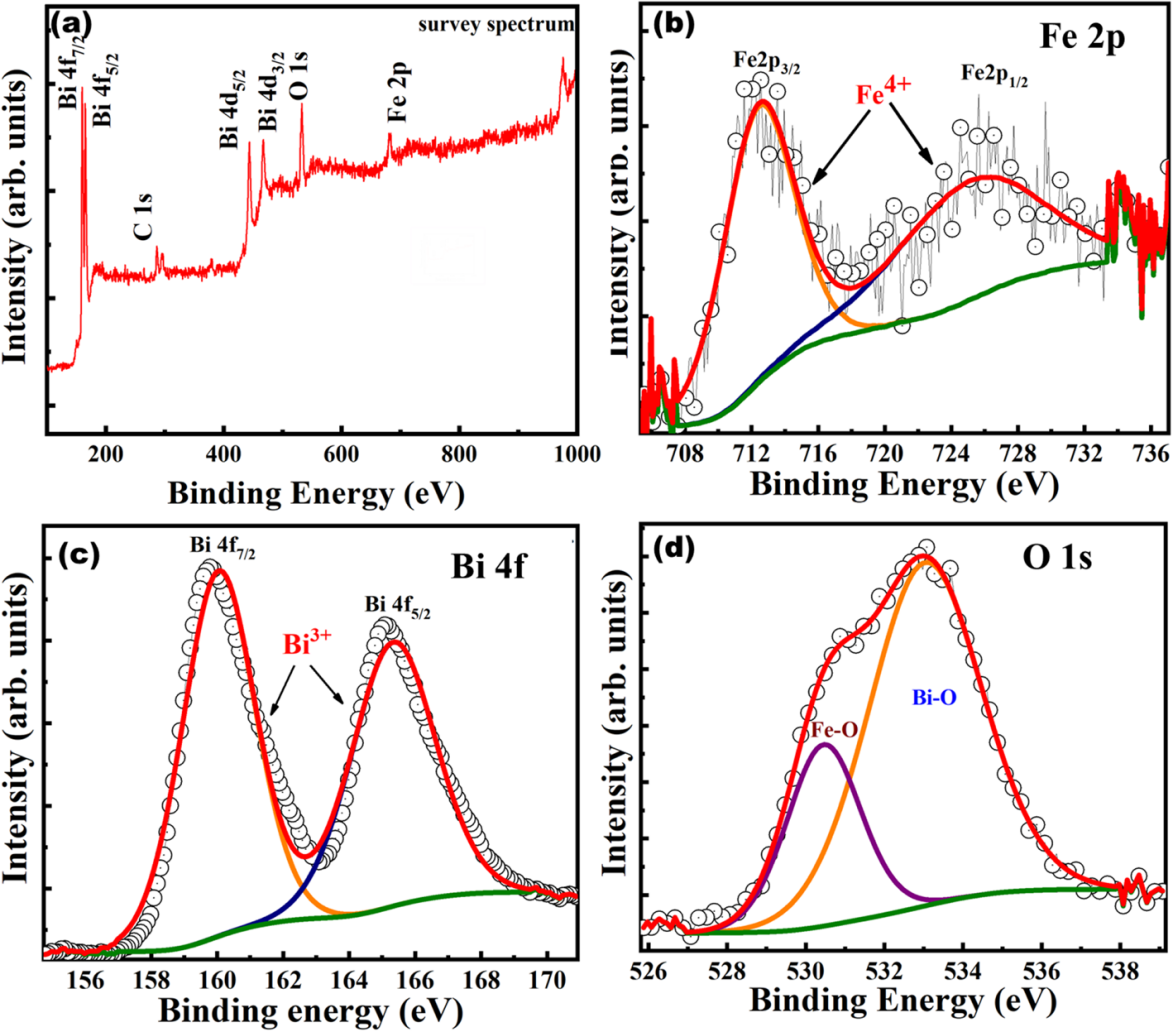
XPS spectra of (**a**) full survey spectrum, (**b**) Fe 2p, (**c**) Bi 4f. and (**d**) O 1s spectra of as synthesized BFO crystals.

**Figure 5:**
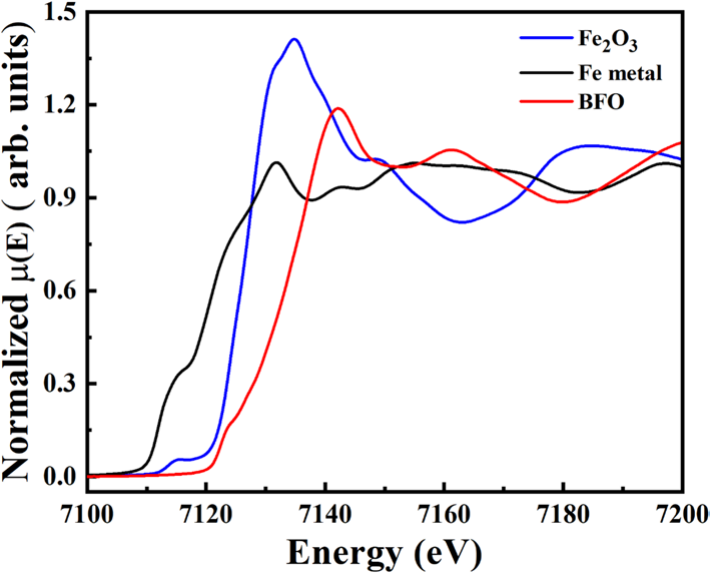
X-ray absorption spectra of Bi_12_FeO_20_ near Fe-K edge position as compared with standard metallic Fe and Fe_2_O_3_.

XPS survey spectrum in Fig. 4a, calibrated using carbon binding energy of 284.6 eV, indicates the presence and oxidation states of constituent Bi, Fe and O elements in Bi_12_FeO_20_ single crystals. XPS has been extensively used to determine the oxidation state of b-site cation, which is expected to possess tetravalency in an ideal sillenite structured Bi_12_FeO_20_. In Fe-based non-ideal sillenite structure such as Bi_12_Fe_0.63_O_18.945_ and Bi_25_FeO_40_, the tetravalency is non-existent^4^. Studies have revealed that Bi_12_Fe_0.63_O_18.945_ constitutes Bi and Fe in  3+ oxidation state, whereas Bi_25_FeO_40_ possesses Bi with multiple oxidation states of 3+ and 5+ and Fe with 3+ oxidation state for charge compensation^25^. The XPS spectra was fitted to Lorentzian-Gaussian utilizing Shirley background method and a spin splitting of 13.4 eV between Fe 2p_3/2_ and Fe 2p_1/2_ was observed corresponding to Fe 2p spectra in Fig. 4b. Fe 2p was fitted to a single peak at 712.6 eV and 726.04 eV for Fe 2p_3/2_ and Fe 2p_1/2_ spin orbits respectively. For Fe 2p_3/2_ the binding energy (B.E) of Fe^2+^ typically exists between 709 eV-710 eV, whereas for Fe^3+^ it exists between 710 eV-711 eV^26^. However, in the present case, it was observed that Fe 2p_3/2_ and Fe 2p_1/2_ correspond to B.Es ~ 712.6 eV and ~ 726.04 eV respectively. The Fe 2p_3/2_and Fe 2p_1/2_peaks shift towards higher energies as compared to typical binding energy of Fe^2+^ and Fe^3+^ oxidation states clearly indicating the existence of higher oxidation state (Fe^4+^) constituting Fe 2p^27–31^. With regard to Bi, the Bi 4f XPS spectra shown in Fig. 4c exhibit two main peaks centered at 165.34 eV (Bi 4f_5/2_) and 160.03 eV (Bi 4f_7/2_) with spin–orbit splitting difference of 5.3 eV corresponding to the binding energy of Bi^+3^^32^. The XPS spectra of O 1s shown in Fig. 4d also revealed two peaks at 530.43 eV and 533.11 eV corresponding to Fe–O and Bi-O bonds respectively^33, 34^. Hence the role of Bi^3+^ and rare occurrence of Fe^4+^ in constituting the ideal Bi_12_FeO_20_ sillenite single crystal was confirmed. The valence state Fe in BFO was also confirmed by XAS studies. The Fe K-edge position of BFO was recorded and compared with standard metallic Fe(0) and Fe_2_O_3_ (Fe^3+^) as shown in Fig. 5. Fe absorption edge for Fe_2_O_3_ was observed at ~ 7120 eV, which corresponds to valence state of Fe^3+^ ^35^. The absorption edge of Fe in BFO is shifted to a higher energy as compared to Fe_2_O_3_, implying that Fe in BFO possesses a higher valence state of Fe^4+^. The XAS studies are in good agreement with XPS studies supporting the existence of Fe with 4+ oxidation state in sillenite Bi_12_FeO_20_.

**Figure 6:**
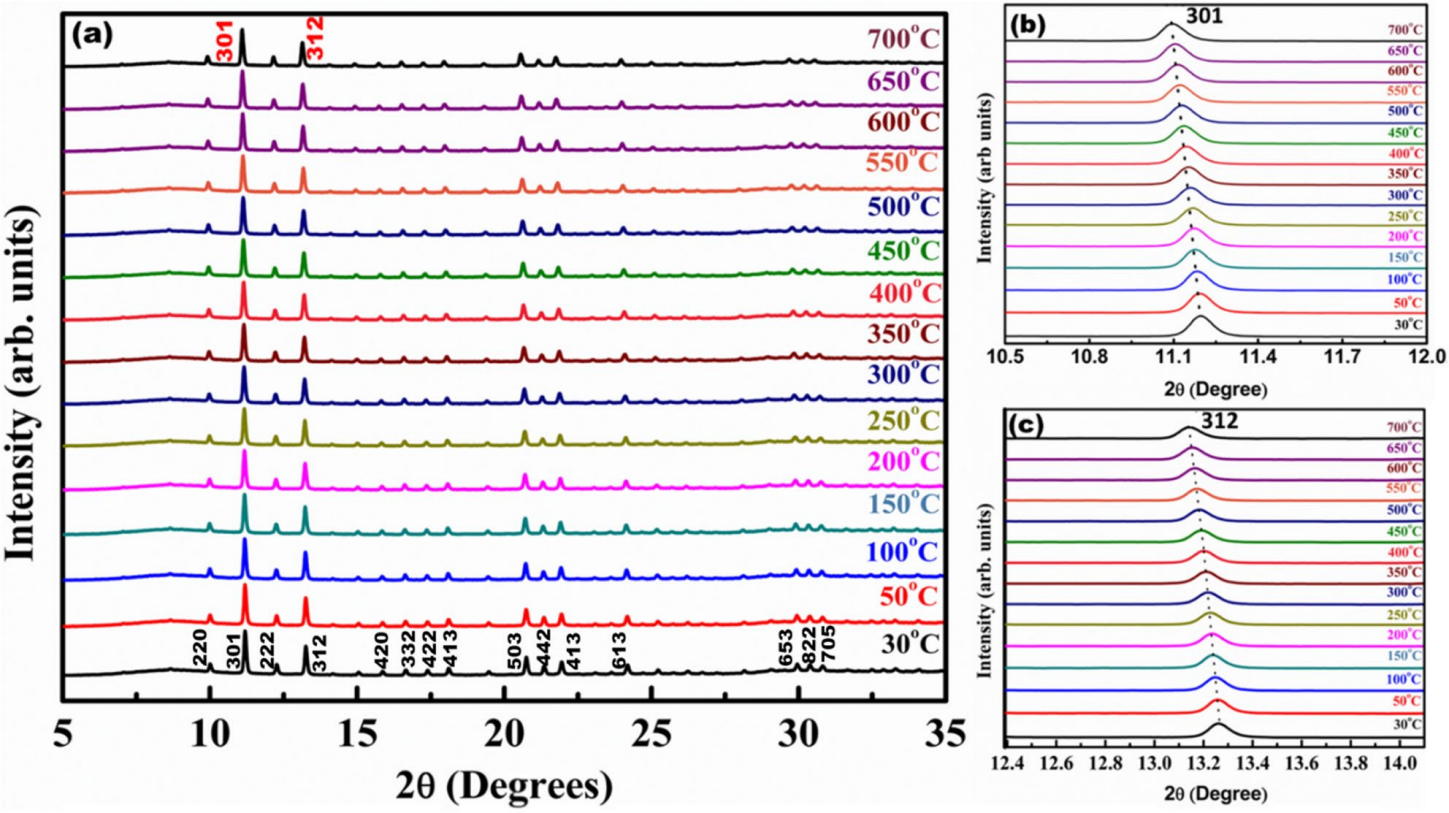
(**a**) Temperature dependent XRD patterns of BFO from 30 °C to 700 °C (**b**,**c**) shift in 2θ with respect to temperature.

Sillenites are also referred to as pyroelectric materials, which can enable them to generate voltages when they experience heat energy. Hence, these materials can be used for thermal sensing devices^36^ and determination of thermal properties of such materials can give new insights into their behavior. Thermal expansion of these materials can be analyzed by finding the coefficient of thermal expansion / thermal expansivity (*α*). Temperature dependent XRD is the one of the accurate methods for determining the molar volume (*V*_*m*_) of a solid because thermal expansion can be calculated per unit cell (atomic level). In the present work, we have attempted to find the thermal expansivity of sillenite BFO using ADXRD within a temperature range of 30–700 °C shown in Fig. 6.

Thermal expansion coefficient (*α*) is the function of change in molar volume (*V*_*m*_) with respect to temperature, as shown below

1 $$\alpha = \frac{1}{{V}_{m}}\left(\frac{\partial {V}_{m}}{\partial T}\right)$$

where *α* is the thermal expansion coefficient, *V*_*m*_ is the molar volume and *T* is the absolute temperature. The value of ‘*α*’ can be determined from the temperature dependent values of *V*_*m*_^37^. Experimentally, this can be done by using temperature dependent XRD to measure the unit cell parameters at various temperatures. For cubic crystal structure materials, the molar volume *V*_*m*_ is defined as

2 $${V}_{m}= \frac{{a}^{3}{N}_{A}}{Z}$$

where *a* is the lattice parameter, *N*_*A*_is the Avogadro’s number (6.022 × 10^23^) and *Z* is the number of formula units per unit cell for body centered cubic cells (*Z* = *2*). Cell parameters, as for example d-spacing (d) and lattice constant, were calculated by Reitveld refinement for XRD patterns recorded at various temperatures. Refined XRD pattern is shown in the supporting information (Figs. S2–S16).

The molar volume *V*_*m*_*,* as a function of temperature can be expressed as

3 $${V}_{m}={V}_{m, 0}\left(1+AT+B{T}^{2}\right),$$

where *V*_*m*,*0 *_is the initial molar volume at 30 °C. Fitting the measured values of *V*_*m*_ in Eq. (3), provides V_m_ as a function of temperature. First order differentiation of Eq. (3) and substitution into Eq. (1) provides the value for thermal expansivity (α).

**Figure 7:**
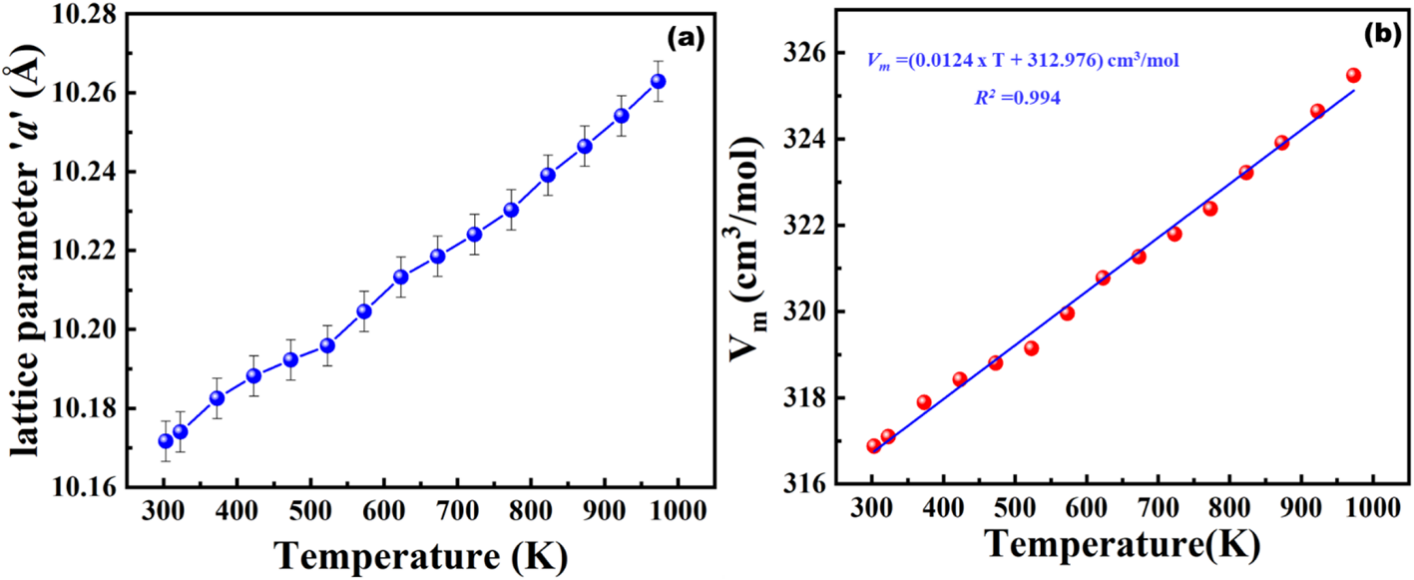
(**a**) The lattice parameter ‘*a*’ as a function of temperature (**b**) molar volume (*V*_*m*_) of BFO as a function of temperature.

From the temperature dependent XRD, we found an increase in lattice parameters and shifting of 2θ value towards lower degree with respect to increase in temperature from 30 °C to 700 °C (Fig. 6). Upon refining the XRD pattern, lattice parameters at corresponding temperatures were calculated. The measured ‘*a’* value was substituted in Eq. (2) and then molar volume (*V*_*m*_) was calculated. The measured ‘*a’* value as the function of temperature is shown in Fig. 7a.

From the fitting of Eq. (3), volumetric changes with respect to temperature was calculated (Fig. 7b) and substituted in Eq. (1). The coefficient of thermal expansion (α) was found to be 3.93 × 10^–5^ K^−1^_._ Differential scanning calorimetry (DSC) measurements (Fig. S18) (in order to determine the phase transition states) were performed up to 1000 °C. Major phase transitions were observed at 770 °C and 827 °C. The endothermic peak at 770 °C and 827 °C might be caused by partial and complete decomposition of the material. Transition above 900 °C is due to melting of decomposed materials.

**Figure 8:**
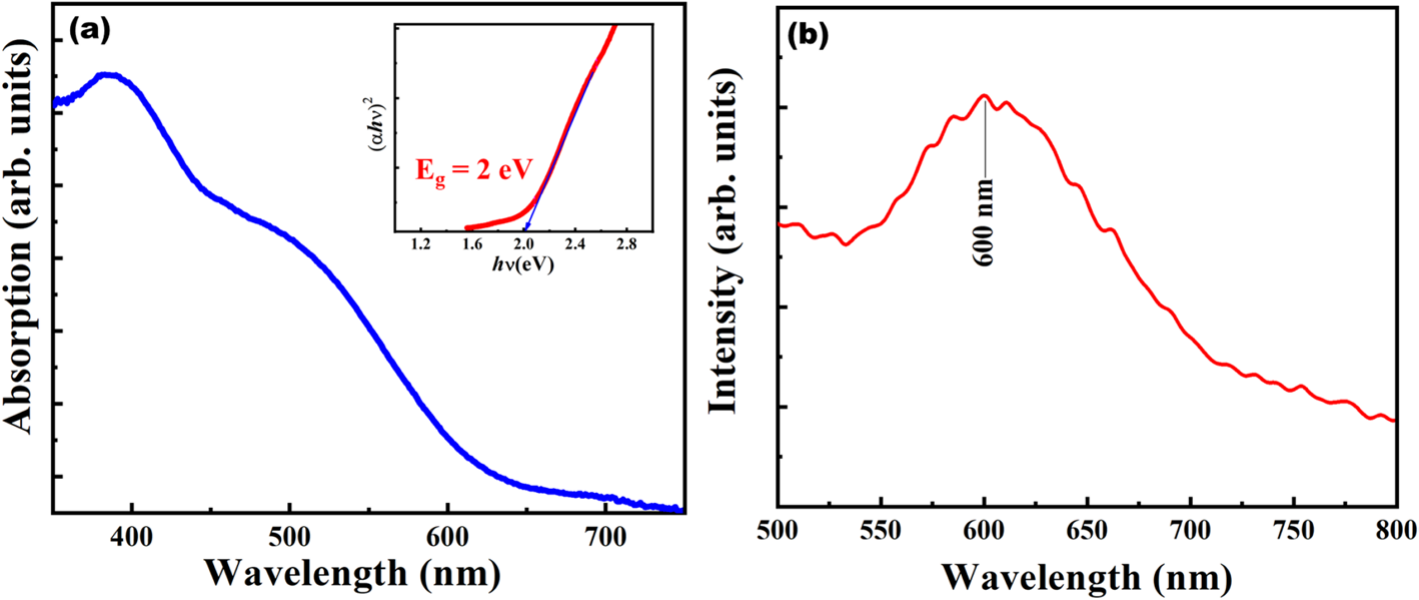
(**a**) Optical absorption spectra (inset Tauc plot to measure bandgap) (**b**) CL spectra of BFO.

The absorption spectra of BFO along with the corresponding Tauc plot (inset Fig. 8a) reveal an effective optical bandgap of 2 eV, which falls under visible region, satisfying a major requirement for photoactive applications like photocatalysis in degradation of organic dyes and water splitting etc.

Any possibility of emission of photons of characteristic wavelengths from ideal Bi_12_FeO_20_ sillenite single crystals can also be probed by Cathodoluminescence (CL) (Fig. 7b) under high-energy electron bombardment. In CL the excitation source can be focused to a probe in an electron microscope providing us with luminescence information having spatial resolution orders of magnitude higher as compared to other techniques. CL on perovskite materials can provide evidence regarding the photoactivity of these materials as it has the potential to resolve emission characteristics in nanoregime^38^. The spatial distribution of emission recorded at 300 K reveals that the entire Bi_12_FeO_20_ crystal radiates predominantly in the visible region. CL spectrum in Fig. 8b obtained at 20 kV for as grown Bi_12_FeO_20_ single crystals, reveals strong band edge emission at about 600 nm (2.04 eV) which is in good agreement with the measured bandgap of BFO single crystals.

**Figure 9:**
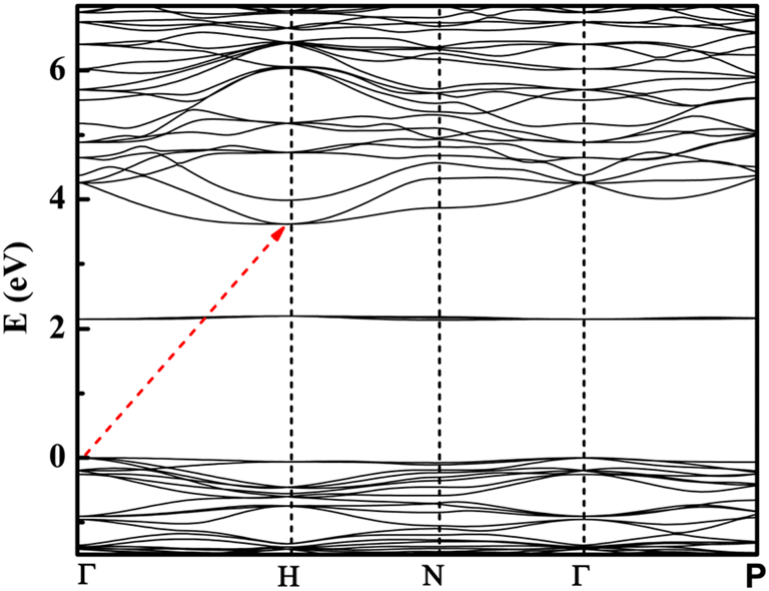
Bandgap energy calculation for sillenite type Bi_12_FeO_20_ (cubic) using density functional calculations.

Further, we compute the electronic band structure shown in Fig. 9. The electronic band structure clearly suggests that Bi_12_FeO_20_ is a wide bandgap indirect semiconductor. The valence band maxima and conduction band minima lie at Γ and H in Brillouin zone with bandgap ~ 3.17 eV, as marked with a red dashed arrow (Fig. 9). The mismatch in experimental and calculated bandgap values is ascribed to superposition. However, it is known that DFT calculations do not reproduce the bandgap values.

**Figure 10:**
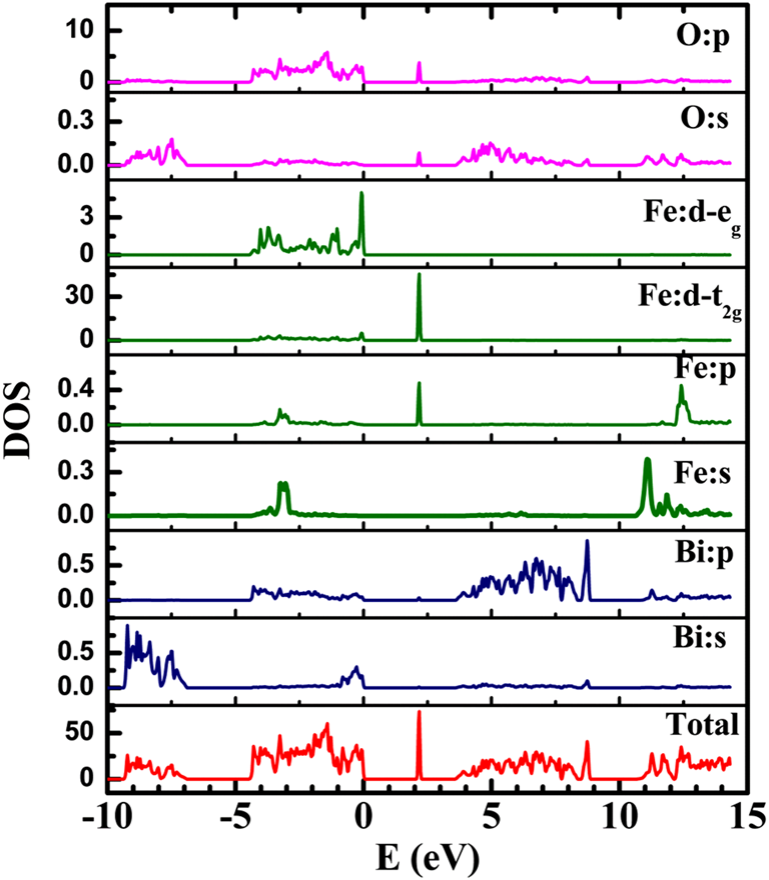
Partial density of Bi (s and p), Fe (s, p, and d) and O (s and p) states in sillenite Bi_12_FeO_20_.

The computed total and partial density of states are plotted in Fig. 10. We find that valence band consists of oxygen (O) s and p orbitals mainly with partial contribution from iron(Fe) s, p and d atomic orbitals, whereas bismuth(Bi) p and oxygen(O) s orbitals contribute mainly to the conduction band. Iron is coordinated as FeO_4_ tetrahedra in Bi_12_FeO_20_ lattice, causing the crystal field splitting in *t*_*2g*_ (*d*_*xy*_*,* *d*_*xz*_ *and* *d*_*xy*_) and *e*_*g*_ (*d*_*x2–y2*_ *and* *d*_*xy*_) orbitals, Fig. 10. The tetrahedral splitting difference is about 2.27 eV with *e*_*g*_ orbitals lying inside the valence band and t_2g_ orbitals lying within the bandgap, giving rise to the intra band states near 2.13 eV, with very large contribution to the density of states. We also computed the absorption spectra of single crysatals (Fig. S18). The large absorption coefficient ~ 10^4^ cm^−1^ is noticed with a small peak ~ 2.13 eV, superimposed with bandgap absorption. This is attributed to the presence of iron intra band states within the bandgap, as noticed in partial density of states, and in experimentally recorded optical absorption spectra of BFO.

**Figure 11:**
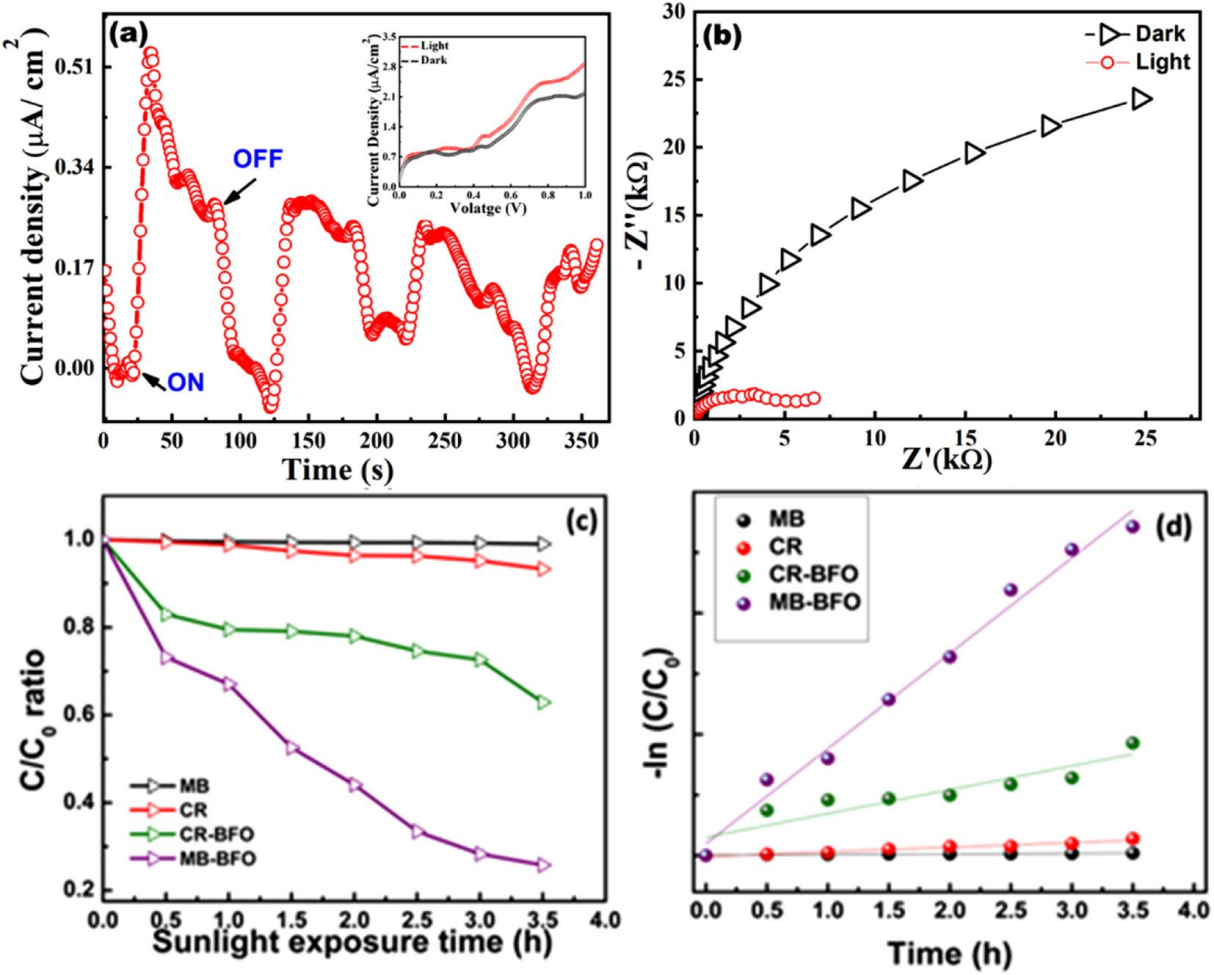
PEC studies on Bi_12_FeO_20_ (**a**) Chronoamperometry curve, shows current response for ON/OFF states of light (inset dark and light I-V characteristics) (**b**) Impedance measurements under dark and light illumination (**c**) C/C_0_ ratio graph of MB and CR dyes with and without catalyst (**d**) First order reaction kinetics for finding rate constant of MB, CR, MB-BFO and CR-BFO under sunlight exposure.

Being a light sensitive compound, sillenite BFO has the potential to act as a photoactive material. A detailed analysis of photoresponse as well as PEC (Photoelectrochemical) measurements can help us to evaluate its photoactive behavior. In the PEC measurement setup, the working electrodes were prepared as follows: BFO crystals were ground to fine powder which was used to form slurry using distilled water. The slurry was coated on FTO and the photoresponse of BFO sample was measured under Xenon lamp source (100 W/cm^2^, AM1.5). Dark and light current measurements at fixed potential (0.5 V) reveal significant photoresponse with proper ON/OFF response observed by chronoamperometry studies Fig. 11a,b. Impedance spectroscopic measurements under dark and light illumination conditions on the sample showed a rapid decrease in impedance under light illumination Fig. 11b. The results imply an efficient and rapid separation of photogenerated charge carriers under light irradiation which leads to photoconductivity as well as a rapid decrease in impedance of the sample.

A direct evidence of photocatalytic activity of as synthesized BFO crystals was found from dye degradation studies carried out with fine ground powder of BFO. BFO was tested to degrade of methylene blue (MB) and Congo red (CR) organic dyes under direct sunlight irradiation. The initial dye concentration of MB and CR was kept at C_o_: 3.5 mg/L and 10 mg/L respectively. The catalyst loaded dye solution was thoroughly blended using ultra sonication under dark condition. The experiment was conducted for different intervals of time under natural sunlight and dyes were found to readily degrade. The sunlight driven degradation of both MB and CR was recorded at regular intervals using UV–visible absorption spectroscopy. After 3.5 h of sunlight exposure MB and CR degraded by 74.23% and 32.10% of their initial concentration respectively. The degradation profile is shown in Fig. S17 along with C/C_0_ ratio graphs and photocatalytic reaction kinetics of MB and CR in Fig. 11c,d. The degradation rate of MB by BFO was found ~0.392 h^−1^, whereas for CR it was ~0.098 h^−1^. Photodegradation kinetics MB and CR in presence of BFO are compared with few reported semiconductor nanoparticles based photocatalyst. BFO showed better performance as compared to some of the existing semiconductor photocatalysts [as tabulated in Table S2] pointing towards the efficacy of BFO as a good photocatalyst material for organic effluent treatment.

## Conclusion

Ideal sillenite spearhead type Bi_12_FeO_20_ single crystals were grown by hydrothermal method for the first time with an average size of ~ 1 mm. The refined single crystal XRD data along with supporting XPS analysis confirms the crystallinity and presence of Bi^3+^ and rare Fe^4+^ oxidation states in BFO respectively. The thermal expansion coefficient was calculated from temperature dependent XRD studies. The bandgap energy from the optical absorption data was found to be ~ 2 eV and the electronic structure was also investigated using first principle calculations. The photoactivity of as grown crystals was proved beyond doubt by PEC measurements, revealing significant photoresponse. CL and photodegradation studies of prepared BFO crystals revealed the luminescence and photocatalytic behavior respectively with promising applications.

## Supplementary information

Supplementary Information 1
