# Author Correction: Growth of sillenite Bi_12_FeO_20_ single crystals: structural, thermal, optical, photocatalytic features and first principle calculations

**DOI:** 10.1038/s41598-021-85663-y

**Published:** 2021-03-17

**Authors:** Durga Sankar Vavilapalli, Ambrose A. Melvin, F. Bellarmine, Ramanjaneyulu Mannam, Srihari Velaga, Himanshu K. Poswal, Ambesh Dixit, M. S. Ramachandra Rao, Shubra Singh

**Affiliations:** 1 Crystal Growth Centre, Anna University, Chennai, 600025 India; 2 University of Bordeaux, ISM UMR CNRS 5255, Bordeaux INP, ENSCBP, 16 Avenue Pey Berland, Bordeaux, 33607 Pessac, France; 3 Nano Functional Materials Technology Centre, Materials Science Research Centre and Department of Physics, Indian Institute of Technology Madras, Chennai, 600036 India; 4 Division of Physics, Department of Science and Humanities, Vignan’s Foundation for Science, Technology and Research, Guntur, 522213 India; 5 High Pressure and Synchrotron Radiation Physics Division, Bhabha Atomic Research Centre, Mumbai, 400085 India; 6 Department of Physics and Centre for Solar Energy, Indian Institute of Technology Jodhpur, Jodhpur, 342 037 India

Correction to: *Scientific Reports* 10.1038/s41598-020-78598-3, published online 16 December 2020

The original version of this Article contained errors in Affiliation 2 and 3, which were incorrectly given as ‘Department of Solar Energy and Environment Physics, Swiss Institute of Dryland Environmental and Energy Research, Jacob Blaustein Institute for Desert Research, Ben-Gurion University of the Negev, 8499000 Midreshet Ben Gurion, Israel’ and ‘University of Bordeaux, ISM UMR CNRS 5255, Bordeaux INP, ENSCBP, 16 Avenue Pey Berland, Bordeaux 33607, Pessac, France’.

The correct affiliations are listed below:

Affiliation 2 ‘University of Bordeaux, ISM UMR CNRS 5255, Bordeaux INP, ENSCBP, 16 Avenue Pey Berland, Bordeaux 33607, Pessac, France’

Affiliation 3 ‘Nano Functional Materials Technology Centre, Materials Science Research Centre and Department of Physics, Indian Institute of Technology Madras, Chennai 600036, India’

These errors have now been corrected in the HTML and PDF versions of the Article.

