# Supplementary material for: Growth of sillenite Bi12FeO20 single crystals: structural, thermal, optical, photocatalytic features and first principle calculations
Source: Sci Rep. 2020 Dec 16;10:22052. doi: 10.1038/s41598-020-78598-3 (PMC7744533; doi:10.1038/s41598-020-78598-3)
Supplement: Supplementary file 1 — Supplementary Information 1 [file 41598_2020_78598_MOESM1_ESM.doc]

**Growth of sillenite Bi_12_FeO_20_ single crystals: Structural, Thermal, Optical, Photocatalytic features and first principle calculations**

Durga Sankar Vavilapalli^1^, Ambrose A. Melvin^2^, Bellarmine F^3^, Ramanjaneyulu Mannam^4^, Velaga Srihari^5^, Himanshu K. Poswal^5^, Ambesh Dixit^6^, M. S. Ramachandra Rao^3^, Shubra Singh ^1*^

*^1^Crystal Growth Centre, Anna University, Chennai, 600025, India.*

*^2^University of Bordeaux, ISM UMR CNRS 5255, Bordeaux INP, ENSCBP16, Avenue Pey Berland, Bordeaux 33607, Pessac, France*

*^3^Nano Functional Materials Technology Centre, Department of Physics, Indian Institute of Technology Madras, Chennai, 600036, India.*

*^4^Division of Physics, Department of Science and Humanities, Vignan’s Foundation for Science, Technology and Research, Guntur 522213, India*

*^5^High Pressure & Synchrotron Radiation Physics Division, Bhabha Atomic Research Centre, 400085, Mumbai, India*

*^6^Department of Physics & Centre for Solar Energy, Indian Institute of Technology Jodhpur, 342 037, Jodhpur, India*

*Email:* [*shubra6@gmail.com*](mailto:shubra6@gmail.com)

***
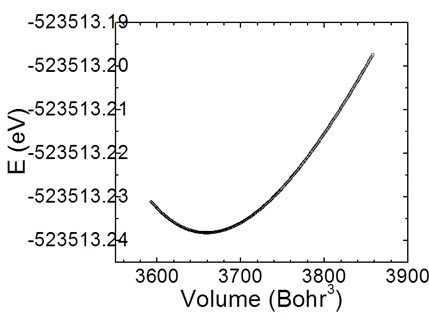
***

Figure S1: Variation of energy versus volume for Bi_12_FeO_20_, used for optimization of lattice parameters.


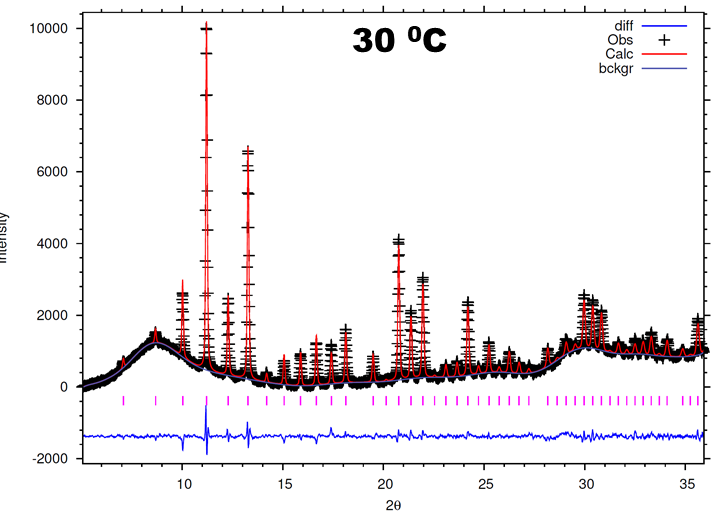
Rietveld refinement analysis of XRD was performed with GSAS.


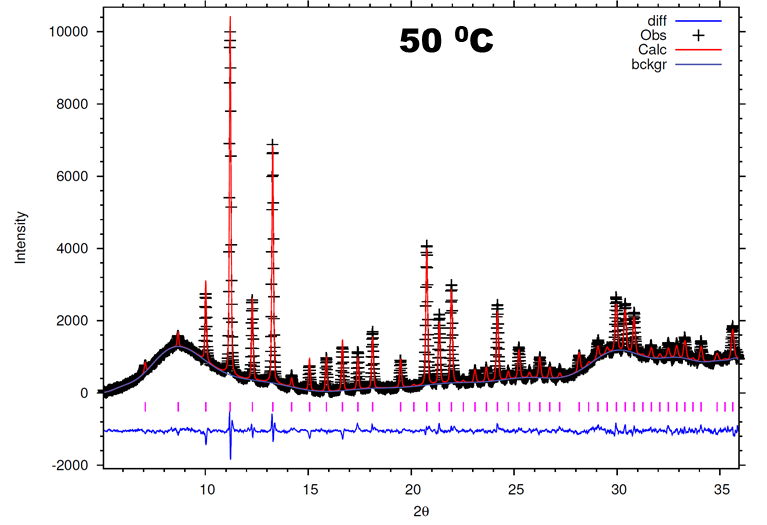
Figure S2: Refined temperature dependent XRD pattern (30^o^C)

Figure S3: Refined temperature dependent XRD pattern (50^o^C)


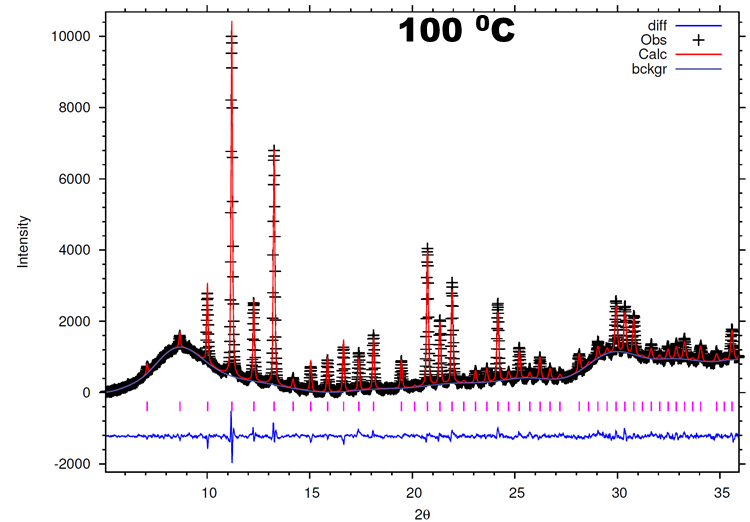


Figure S4: Refined temperature dependent XRD pattern (100^o^C)


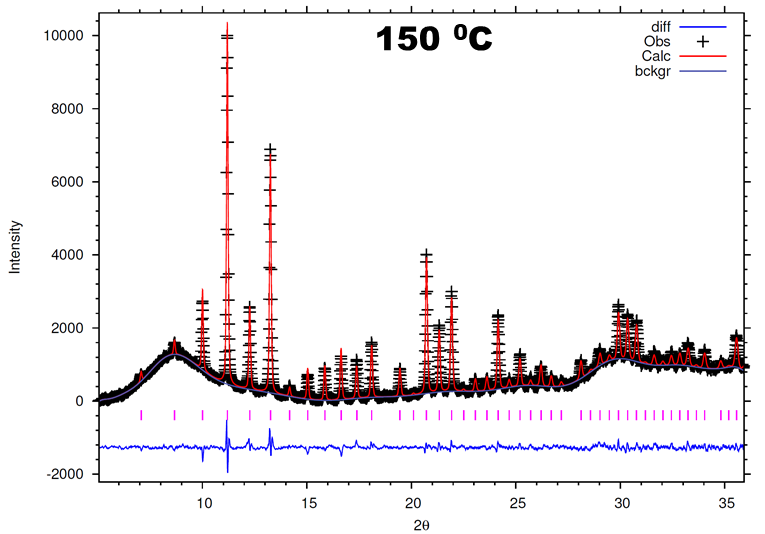


Figure S5: Refined temperature dependent XRD pattern (150^o^C)


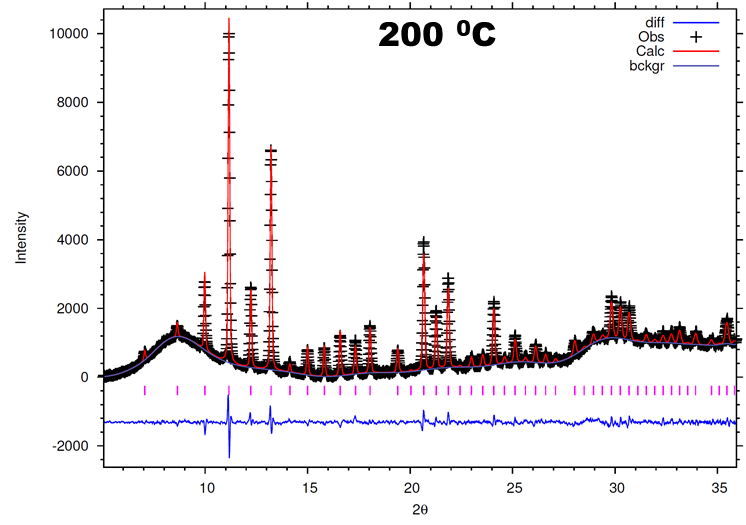


Figure S6: Refined temperature dependent XRD pattern (200^o^C)


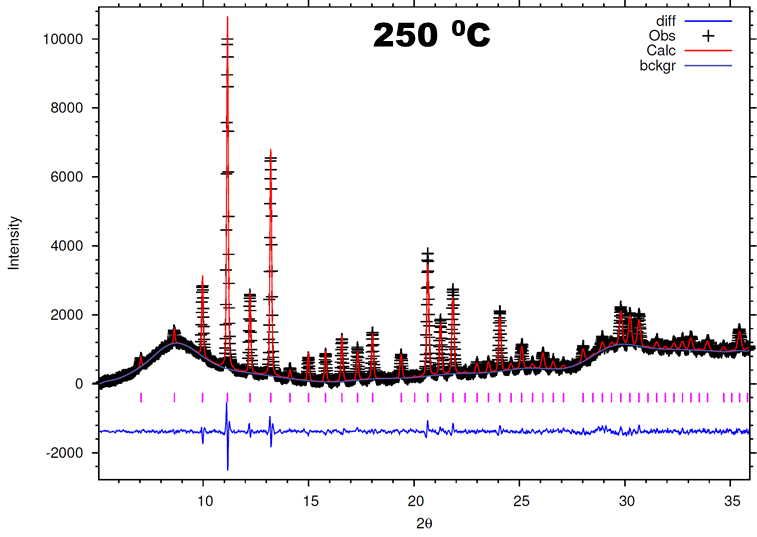


Figure S7: Refined temperature dependent XRD pattern (250^o^C)


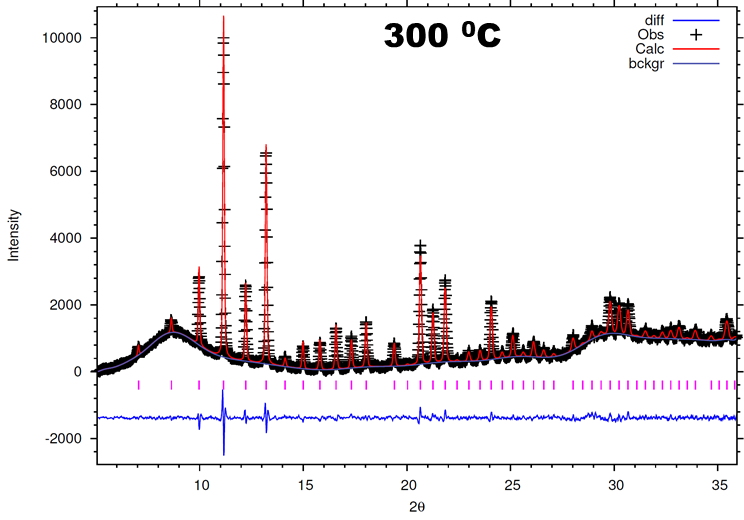


Figure S8: Refined temperature dependent XRD pattern (300^o^C)


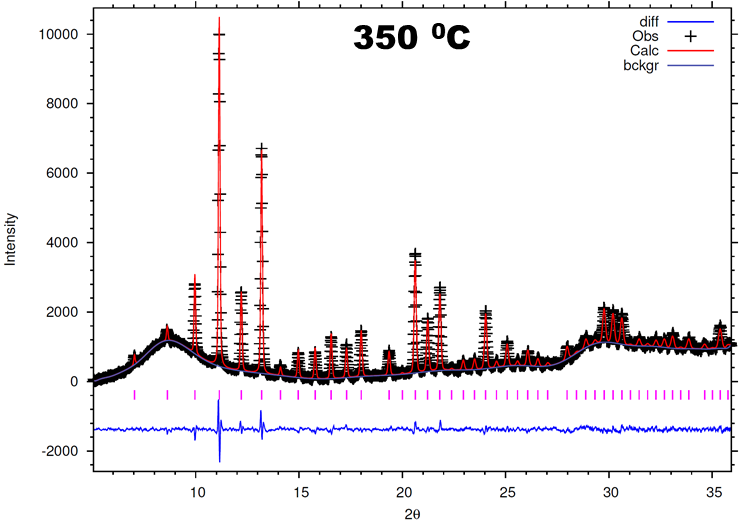


Figure S9: Refined temperature dependent XRD pattern (350^o^C)


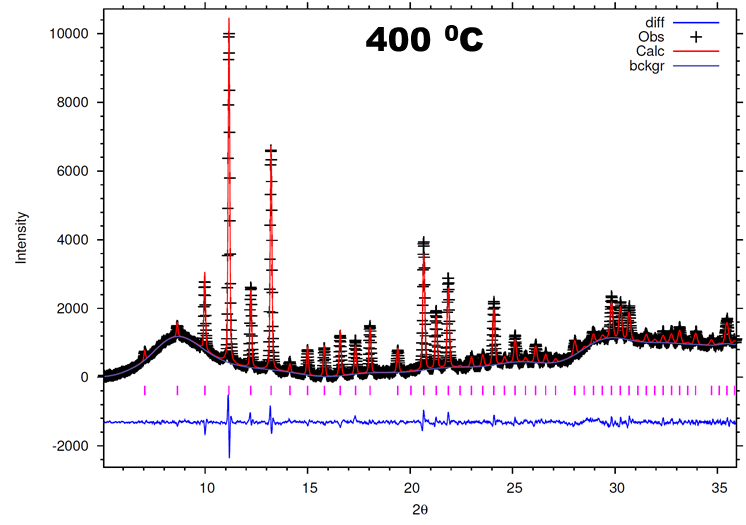


Figure S10: Refined temperature dependent XRD pattern (400^o^C)


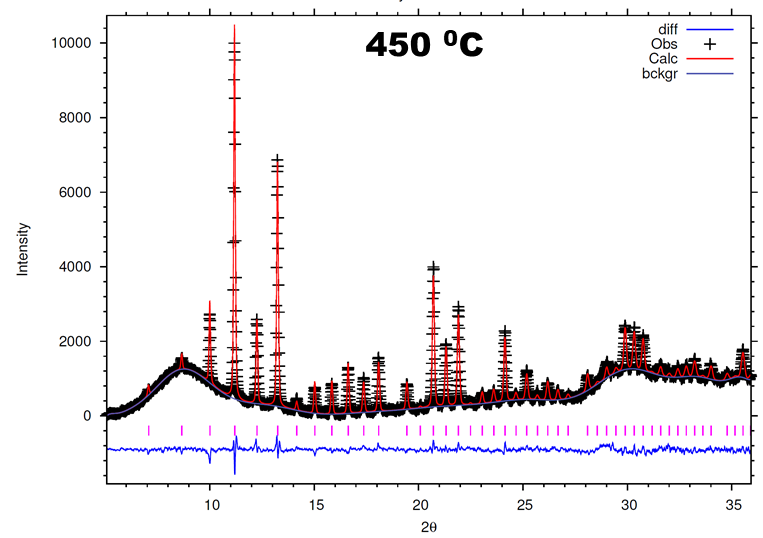


Figure S11: Refined temperature dependent XRD pattern (450^o^C)


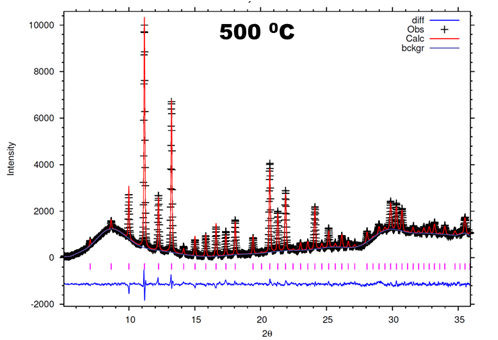


Figure S12: Refined temperature dependent XRD pattern (500^o^C)


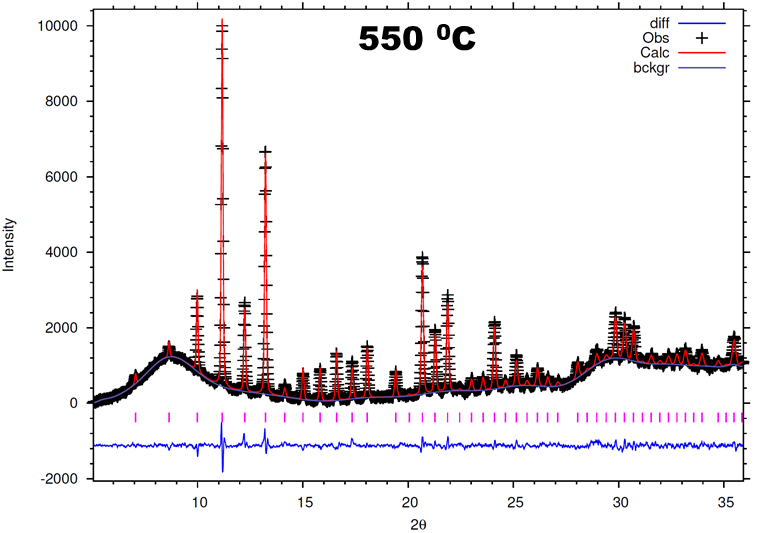


Figure S13: Refined temperature dependent XRD pattern (550^o^C)


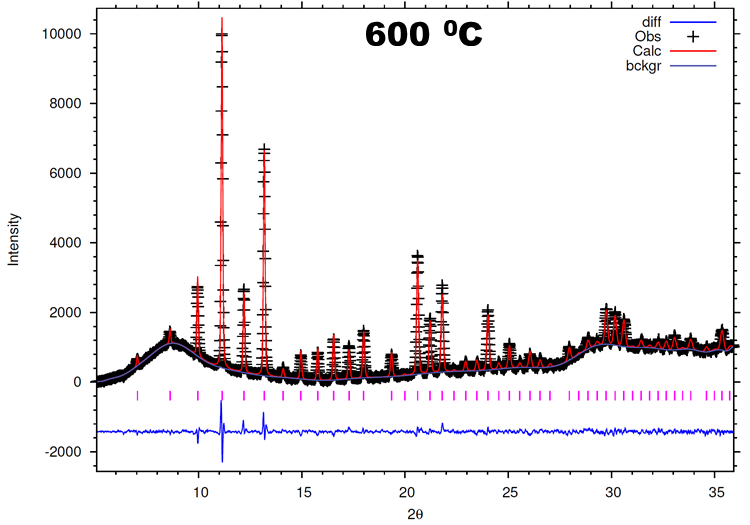


Figure S14: Refined temperature dependent XRD pattern (600^o^C)


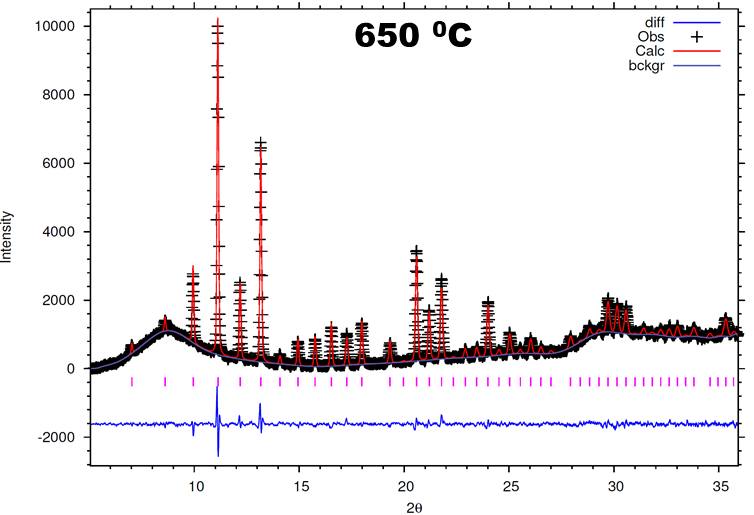


Figure S15: Refined temperature dependent XRD pattern (650^o^C)


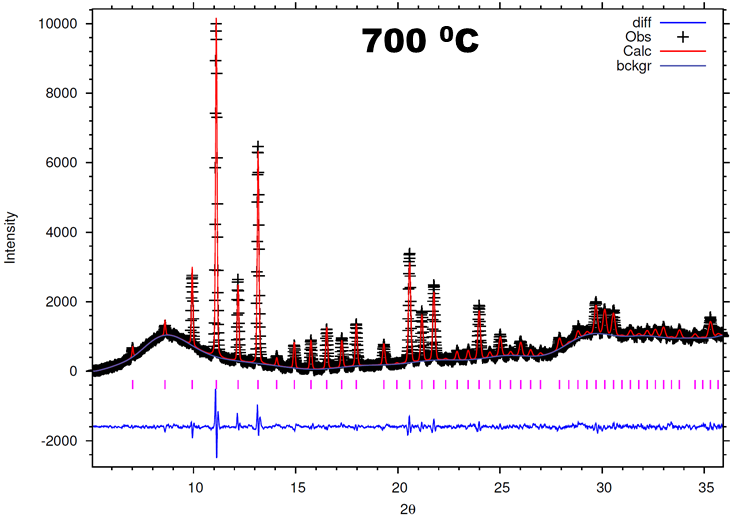


Figure S16: Refined temperature dependent XRD pattern (700^o^C)


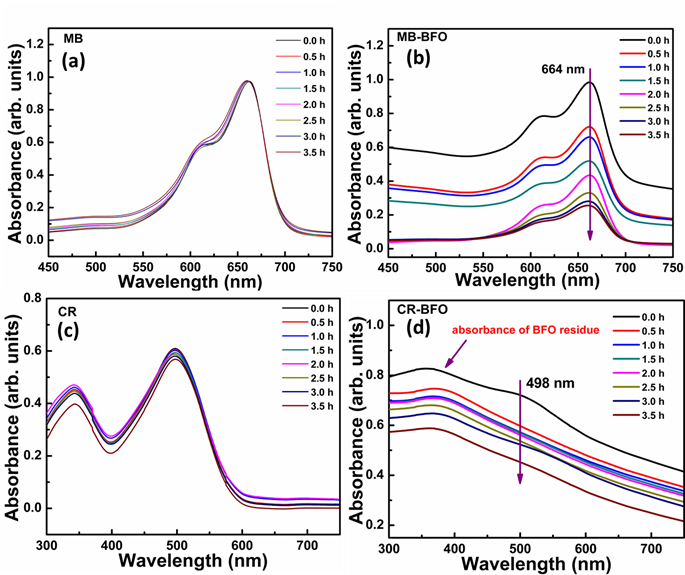


Figure S17: (a) Photocatalytic degradation of MB without BFO particulates (b) Photocatalytic degradation of MB by BFO particulates (c) Photocatalytic degradation of CR without BFO particulates (d) Photocatalytic degradation of CR by BFO particulates


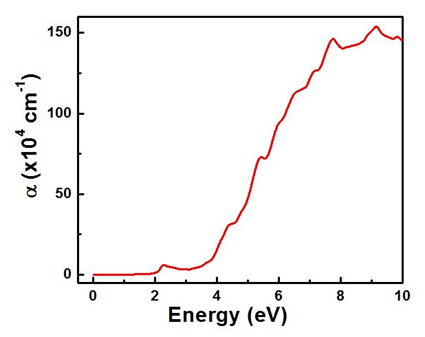


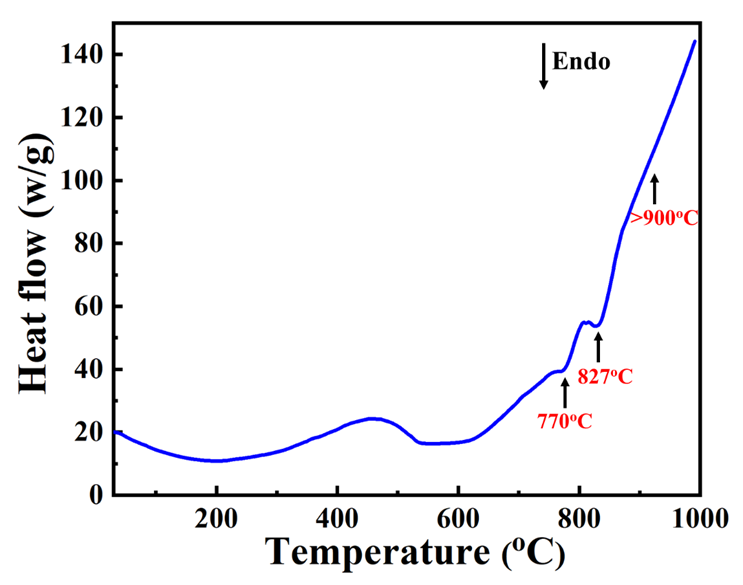
Figure S18: Absorption coefficient for nonmagnetic Bi_12_FeO_20_ system

Figure S19: Thermal analysis results of Bi_12_FeO_20_ sample using DSC

Figure:S19 shows the DSC curve of Bi_12_FeO_20_, where the major phase transitions were observed at 770^o^C and 827^o^C. The endothermic peak at 770^o^C and 827^o^C might be caused by partial and complete decomposition of the material. Transition above 900^o^C is due to melting of decomposed materials [1].

1. Chen, Y.; Wu, Q.; Zhao, J., Selective synthesis on structures and morphologies of BixFeyOz nanomaterials with disparate magnetism through time control. Journal of Alloys and Compounds 2009, 487 (1), 599-604.

**Table S1.** Single crystal structure refinement data of as grown Bi_12_FeO_20_ single crystals

| Empirical formula | Bi_12_FeO_20_ |
| --- | --- |
| Formula weight | 2883.61 |
| Temperature | 296(2) K |
| Wavelength | 0.71073 Å |
| Crystal system | Cubic |
| Space group | I23 |
| Unit cell dimensions | a = 10.1713(1) Å α = 90° b = 10.1713(1) Å β= 90° c = 10.1713(1) Å γ= 90° |
| Volume | 1052.28(3) Å^3^ |
| Z | 2 |
| Calculated density | 9.101 Mg/m^3^ |
| Absorption coefficient | 100.685 mm^-1^ |
| F(000) | 2364 |
| Crystal size | 0.200 x 0.150 x 0.100 mm^3^ |
| Theta range for data collection | 2.832 to 24.971°. |
| Index ranges | -12<=h<=12, -12<=k<=11, -12<=l<=8 |
| Reflections collected | 5484 |
| Independent reflections | 322 [R(int) = 0.1116] |
| Completeness to theta = 24.971° | 100.0 % |
| Absorption correction | Semi-empirical from equivalents |
| Max. and min. transmission | 0.724 and 0.204 |
| Refinement method | Full-matrix least-squares on F^2^ |
| Data / restraints / parameters | 322 / 0 / 27 |
| Goodness-of-fit on F^2^ | 1.124 |
| Final R indices [I > 2sigma(I)] | R1 = 0.0236, wR2 = 0.0553 |
| R indices (all data) | R1 = 0.0255, wR2 = 0.0564 |
| Absolute structure parameter | 0.04(3) |
| Extinction coefficient | 0.00035(6) |
| Largest diff. peak and hole | 1.324 and -1.490 e Å^-3^ |

Atomic coordinates ( x 10^4^) and equivalent isotropic displacement parameters (Å^2^x 10^3^)

for i23. U(eq) is defined as one third of the trace of the orthogonalized Uij tensor.

________________________________________________________________________________

x y z U(eq)

________________________________________________________________________________

Bi(1) 3239(1) 1822(1) 5153(1) 14(1)

Fe(1) 5000 5000 5000 13(2)

O(1) 1355(14) 2522(14) 4877(16) 13(4)

O(2) 3079(15) 3079(15) 6921(15) 14(6)

O(3) 3943(19) 3943(19) 3943(19) 36(10)

________________________________________________________________________________

Bond lengths [Å] and angles [°] for i23.

_____________________________________________________

Bi(1)-O(1) 2.064(14)

Bi(1)-O(2) 2.213(5)

Bi(1)-O(1)#1 2.254(17)

Bi(1)-O(3) 2.585(12)

Bi(1)-O(1)#2 2.620(15)

Bi(1)-Bi(1)#3 3.5951(17)

Fe(1)-O(3)#4 1.86(3)

Fe(1)-O(3)#5 1.86(3)

Fe(1)-O(3)#3 1.86(3)

Fe(1)-O(3) 1.86(3)

O(1)-Bi(1)#6 2.254(17)

O(1)-Bi(1)#7 2.620(15)

O(2)-Bi(1)#8 2.213(5)

O(2)-Bi(1)#9 2.213(5)

O(3)-Bi(1)#10 2.585(12)

O(3)-Bi(1)#11 2.585(12)

O(1)-Bi(1)-O(2) 80.9(5)

O(1)-Bi(1)-O(1)#1 91.1(2)

O(2)-Bi(1)-O(1)#1 88.7(7)

O(1)-Bi(1)-O(3) 84.5(5)

O(2)-Bi(1)-O(3) 85.7(8)

O(1)#1-Bi(1)-O(3) 173.3(5)

O(1)-Bi(1)-O(1)#2 84.65(14)

O(2)-Bi(1)-O(1)#2 152.9(7)

O(1)#1-Bi(1)-O(1)#2 68.8(6)

O(3)-Bi(1)-O(1)#2 115.7(7)

O(1)-Bi(1)-Bi(1)#3 156.0(4)

O(2)-Bi(1)-Bi(1)#3 98.2(4)

O(1)#1-Bi(1)-Bi(1)#3 112.9(4)

O(3)-Bi(1)-Bi(1)#3 71.5(3)

O(1)#2-Bi(1)-Bi(1)#3 104.1(3)

O(3)#4-Fe(1)-O(3)#5 109.471(2)

O(3)#4-Fe(1)-O(3)#3 109.471(6)

O(3)#5-Fe(1)-O(3)#3 109.471(1)

O(3)#4-Fe(1)-O(3) 109.471(1)

O(3)#5-Fe(1)-O(3) 109.471(3)

O(3)#3-Fe(1)-O(3) 109.471(2)

Bi(1)-O(1)-Bi(1)#6 119.3(7)

Bi(1)-O(1)-Bi(1)#7 109.8(6)

Bi(1)#6-O(1)-Bi(1)#7 99.2(5)

Bi(1)#8-O(2)-Bi(1)#9 116.9(4)

Bi(1)#8-O(2)-Bi(1) 116.9(4)

Bi(1)#9-O(2)-Bi(1) 116.9(4)

Fe(1)-O(3)-Bi(1)#10 111.5(7)

Fe(1)-O(3)-Bi(1)#11 111.5(7)

Bi(1)#10-O(3)-Bi(1)#11 107.3(7)

Fe(1)-O(3)-Bi(1) 111.5(7)

Bi(1)#10-O(3)-Bi(1) 107.3(7)

Bi(1)#11-O(3)-Bi(1) 107.3(7)

_____________________________________________________________

Symmetry transformations used to generate equivalent atoms:

#1 -y+1/2,-z+1/2,x+1/2 #2 -y+1/2,z-1/2,-x+1/2

#3 -x+1,y,-z+1 #4 -x+1,-y+1,z #5 x,-y+1,-z+1

#6 z-1/2,-x+1/2,-y+1/2 #7 -z+1/2,-x+1/2,y+1/2

#8 -z+1,x,-y+1 #9 y,-z+1,-x+1 #10 y,z,x #11 z,x,y

Anisotropic displacement parameters (Å^2^x 10^3^) for i23. The anisotropic

displacement factor exponent takes the form: -2π2[ h^2^a*^2^U^11^ + ... + 2 h k a* b* U^12^ ]

______________________________________________________________________________

U^11^ U^22^ U^33^ U^23^ U^13^ U^12^

______________________________________________________________________________

Bi(1) 8(1) 20(1) 14(1) -5(1) 2(1) 3(1)

Fe(1) 13(2) 13(2) 13(2) 0 0 0

O(1) 9(8) 12(9) 18(9) -3(8) -3(7) 2(6)

O(2) 14(6) 14(6) 14(6) -9(8) -9(8) 9(8)

O(3) 36(10) 36(10) 36(10) -8(10) -8(10) -8(10)

______________________________________________________________________________

**Table S2.** A comparison of the photocatalytic activity of BFO with some of the existed semiconductor photocatalysts

| **S.No** | **Catalyst** | **Dye concentration and catalyst loading** | **%degradation and degradation time** | **Rate constant**  **(h^-1^)** | **Light source** | **Ref.** |
| --- | --- | --- | --- | --- | --- | --- |
| 1 | Bi_12_FeO_20_ | MB(3.5mg/L), 0.25g/L  CR(10mg/L), 0.25g/L | 74.23% (3hr 30 min)  32.10% (3hr 30 min) | 0.3929  0.098 | Sun light | This work |
| 2 | TiO2-P25  N-TiO2  Yb-TiO2 | MB(10mg/L),0.15g/50ml  MB(10mg/L),0.15g/50ml  MB(10mg/L),0.15g/50ml | 45.72% (5h)  _  _ | 0.12143  0.29908  0.26324 | Vis. Light  30W fluorescent lamp  400-750 nm | [^1^](#_ENREF_1) |
| 3 | PEDOT  PEDOT/GO | MB(-), 20mg/50ml  MB(-), 20mg/50ml | 21.2% (7h)  72.9% (7h) | -  - | Sun light | [^2^](#_ENREF_2) |
| 4 | ZnS  CdS  ZnS/CdS | MB(10mg/L), 100mg/L | 30%(6h)  63%(6h)  65% (6h) | 0.0654  0.1788  0.1734 | Vis light  500W  Halogen lamp  400-800nm | [^3^](#_ENREF_3) |
| 5 | Sb-CdS  CdS | MB(8ppm), 50mg/200ml | 70%(6h)  68%(6h) | 0.18  0.114 | Vis. light | [^4^](#_ENREF_4) |
| 6 | SnO2 | CR(0.034mM), 2mg/25ml | 20% (4h) | - | 230V, UV lamp | [^5^](#_ENREF_5) |
| 7 | NiO Nanoparticles  NiO Nanofibers | CR(60 ppm), 60mg  CR(60 ppm), 60mg | 50%(8h)  70%(8h) | 0.0067  0.0037 | Vis. light | [^6^](#_ENREF_6) |

**References:**

1. Zhang, J.; Xu, L. J.; Zhu, Z. Q.; Liu, Q. J., Synthesis and properties of (Yb, N)-TiO_2_ photocatalyst for degradation of methylene blue (MB) under visible light irradiation. *Materials Research Bulletin* **2015**, *70*, 358-364.

2. Zhang, L.; Jamal, R.; Zhao, Q.; Wang, M.; Abdiryim, T., Preparation of PEDOT/GO, PEDOT/MnO_2_, and PEDOT/GO/MnO_2_ nanocomposites and their application in catalytic degradation of methylene blue. *Nanoscale Research Letters* **2015**, *10* (1), 148.

3. Soltani, N.; Saion, E.; Hussein, M. Z.; Erfani, M.; Abedini, A.; Bahmanrokh, G.; Navasery, M.; Vaziri, P., Visible Light-Induced Degradation of Methylene Blue in the Presence of Photocatalytic ZnS and CdS Nanoparticles. *International Journal of Molecular Sciences* **2012**, *13* (10), 12242.

4. Ertis, I. F.; Boz, I., Synthesis and Characterization of Metal-Doped (Ni, Co, Ce, Sb) CdS Catalysts and Their Use in Methylene Blue Degradation under Visible Light Irradiation. *Modern Research in Catalysis* **2017**, *Vol.06No.01*, 14.

5. Kar, A.; Kundu, S.; Patra, A., Photocatalytic properties of semiconductor SnO2/CdS heterostructure nanocrystals. *RSC Advances* **2012**, *2* (27), 10222-10230.

6. Malwal, D.; Gopinath, P., Fabrication and characterization of poly(ethylene oxide) templated nickel oxide nanofibers for dye degradation. *Environmental Science: Nano* **2015**, *2* (1), 78-85.
